# UCell and pyUCell: single-cell gene signature scoring for R and Python

**DOI:** 10.1093/bioinformatics/btag055

**Published:** 2026-02-03

**Authors:** Massimo Andreatta, Santiago J Carmona

**Affiliations:** Department of Pathology and Immunology, Faculty of Medicine, University of Geneva, Geneva 1206, Switzerland; Translational Research Centre in Onco-Hematology (CRTOH), Geneva 1211, Switzerland; Geneva Centre for Inflammation Research (GCIR), Geneva 1211, Switzerland; Swiss Institute of Bioinformatics, Lausanne 1015, Switzerland; Department of Pathology and Immunology, Faculty of Medicine, University of Geneva, Geneva 1206, Switzerland; Translational Research Centre in Onco-Hematology (CRTOH), Geneva 1211, Switzerland; Geneva Centre for Inflammation Research (GCIR), Geneva 1211, Switzerland; Swiss Institute of Bioinformatics, Lausanne 1015, Switzerland

## Abstract

**Summary:**

Gene signature scoring provides a simple yet powerful approach for quantifying biological signals within single-cell omics datasets. UCell and pyUCell offer fast and robust implementations of rank-based signature scoring for R and Python, respectively, integrating seamlessly with leading single-cell analysis ecosystems such as Seurat, Bioconductor, and scanpy/scverse.

**Availability and implementation:**

UCell v2 is distributed as an R package by BioConductor (https://bioconductor.org/packages/UCell/) and as a Python package by pyPI (https://pypi.org/project/pyucell/).

## 1 Introduction

Single-cell technologies have transformed our ability to characterize cellular heterogeneity by profiling gene expression at the resolution of individual cells. A central computational task in single-cell analysis is quantifying the activity of predefined gene sets (or “modules”) within individual cells–an approach commonly referred to as gene signature scoring or module scoring. Gene sets can be derived from a variety of sources, including curated pathway databases such as MSigDB, Reactome, or KEGG (Liberzon *et al.* 2015, Kanehisa *et al.* 2017, Milacic *et al.* 2024); literature-defined marker panels describing specific cell types or activation states; and data-driven approaches in which modules are inferred from co-expression networks, differential expression analyses, or latent factors modeling (Aibar *et al.* 2017, Stein-O’Brien *et al.* 2018, Morabito *et al.* 2023). In single-cell studies, such gene sets are routinely used to annotate cell clusters, identify activation programs, and quantify pathway activity at the single-cell level (Luecken and Theis 2019).

To provide a robust and scalable solution for single-cell gene signature scoring, we previously introduced UCell, a method based on the Mann–Whitney U statistic. UCell’s rank-based scoring framework ensures robustness to dataset size and heterogeneity, while enabling efficient computation in both memory and runtime (Andreatta and Carmona 2021). Since its introduction, UCell has become a widely adopted tool in the single-cell community for reproducible and computationally efficient quantification of gene signature activity. For alternative state-of-the-art signature scoring tools, we refer readers to recent reviews and benchmarks (Noureen *et al.* 2022, Fan *et al.* 2024, Wang and Thakar 2024). In this work, we present algorithmic updates and extensions to UCell (v2), including support for positive and negative gene sets, improved handling of missing genes, and the option to smooth signature scores using nearest-neighbor relationships in gene expression space. We discuss key parameters of the method and introduce pyUCell, a native Python implementation designed for seamless integration into common single-cell analysis ecosystems such as Scanpy (Wolf, Angerer and Theis 2018) and scverse (Virshup *et al.* 2023).

## 2 Implementation

### 2.1 Input formats

The essential input to UCell is a gene-by-cell expression matrix, which may originate from single-cell RNA sequencing (scRNA-seq), spatial transcriptomics, or other single-cell modalities. UCell scores can be calculated directly on raw counts or on normalized expression data (see Supplementary Note, available as supplementary data at *Bioinformatics* online for more details). UCell v2 supports multiple input formats, including SingleCellExperiment and Seurat objects (for R), as well as AnnData (for Python).

### 2.2 UCell score calculation

Given a (genes by cells) gene expression matrix **M** and a gene signature **s** = (s_1_, s_2_, …, s_*n*_) composed of *n* genes, we calculate UCell scores independently for each cell using:


$$U\mathrm{Cell}=1-\frac{U}{U_{\max}}$$

where the Mann–Whitney U statistic (Mann and Whitney 1947) is calculated with:


$$U=\sum_{i=1}^{n}r_{i}-\frac{n(n+1)}{2}$$

and the normalization factor *U_max_* is:


$$U_{\max}=n\cdot r_{\max}-\frac{n(n+1)}{2}$$

The vector **r** = (r_1_, r_2_, …, r_*n*_) contains the relative ranks of the genes in signature **s** = (s_1_, s_2_, …, s_n_) in column **M_:,j_** of the gene expression matrix, corresponding to a vector of expression values for cell *j*. To account for data sparsity, rank values are capped at the maximum rank value r_max__*:*_


$$r_{i}\leftarrow \min\left(r_{i},\;r_{\max}\right),\;i=1,\ldots ,n$$

See below for a discussion of the *r_max_* parameter. We note that the first implementation of the UCell algorithm used *U_max_* = *n ⋅ r_max_*_,_ which overestimated the theoretical maximum value for the Mann-Whitney U statistic.

### 2.3 Positive and negative gene sets

**Figure 1:**
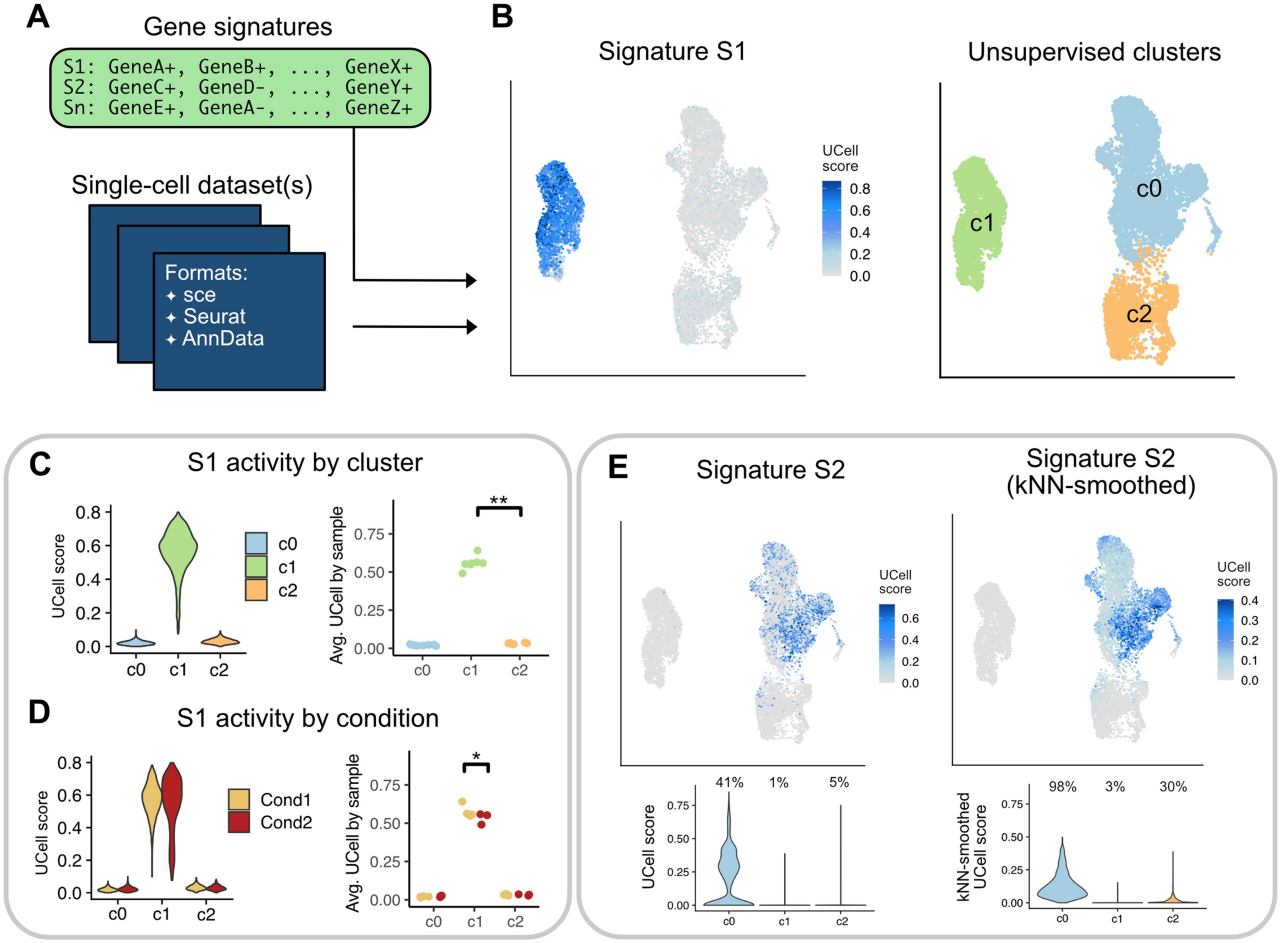
Signature scoring with UCell. (A) Gene signatures (comprising both positive and negative gene sets) are evaluated for each cell in one or several single-cell datasets; several input formats are accepted. (B) Gene signatures can be visualized in low-dimensional spaces and quantified in different groupings of the data, e.g. unsupervised cell clusters. (C) Distribution of UCell scores for a representative signature (S1) in three unsupervised clusters (c0, c1, c2) at the single-cell level (left) or aggregated by sample (right). (D) Distribution of UCell scores for a representative signature (S1) in three unsupervised clusters (c0, c1, c2) and two experimental conditions (Cond1, Cond2) at the single-cell level (left) or aggregated by sample (right). (E) kNN-smoothing for a representative signature (S2), exemplifying the removal of outlier values and reduction of zeros in UCell score distributions. Values above the violins indicate the percentage of non-zero scores in each distribution.

A major enhancement to UCell since its initial release is the ability to incorporate both positive and negative gene sets within a single signature. Given two gene sets, **s^+^** and **s^-^**, UCell scores are computed independently for each set and then combined as follows:


$$\text{UCell}={\text{UCell}}^{+}-w\cdot {\text{UCell}}^{-}$$

where *w* is a weighting parameter (*w = 1* by default) controlling the relative contribution of the negative component. The resulting combined score is clipped at zero to ensure that UCell values remain within the [0,1] range. In practice, positive and negative gene sets are provided to the algorithm as a single signature, with each gene labeled using a + or – suffix, following standard conventions in the biological literature (Fig. 1A).

### 2.4 The *r*_max_ parameter

Single-cell omics datasets are typically sparse, with a substantial fraction of zero expression values. Consequently, gene rankings are only meaningful within the range of non-zero measurements. To prevent the spurious ranking of a large tail of zeros, UCell caps rank values at a maximum threshold defined by the parameter *r_max_*. As a practical guideline, *r_max_* should be set approximately to the median number of detected (non-zero) genes per cell. This choice ensures that rankings focus on the informative portion of each cell’s expression profile. The default *r_max_* = 1500 is suitable for 10X Chromium scRNA-seq, which is the most common application. The selection of *r_max_* is particularly important for datasets with limited feature space. For example, probe-based spatial transcriptomics platforms (e.g. Xenium, CosMx) typically measure a few hundred to a few thousand genes, while antibody-derived tag (ADT) data in CITE-seq experiments usually include up to a few hundred proteins. The *r_max_* parameter should therefore be adjusted according to the dimensionality of the experiment. When comparing multiple samples, it is recommended to set the same value of *r_max_* for all samples (see also Supplementary Note, available as supplementary data at *Bioinformatics* online).

### 2.5 Handling missing genes

An important technical consideration is how to handle features (e.g. signature genes) that are absent from the input matrix. UCell v2 offers two options for dealing with missing genes, reflecting different assumptions about the data. *Option 1—impute missing genes as zero counts:* assumes that genes absent from the count matrix have zero (or below-detection) expression in the cell. This situation commonly arises in processed scRNA-seq datasets deposited in public repositories, where lowly detected genes are often filtered out during preprocessing. Under this assumption, missing genes are treated as unexpressed and contribute accordingly to the signature score. *Option 2—skip missing genes:* assumes that missing genes were not measured by the assay rather than being unexpressed. This option should be selected for targeted or panel-based single-cell technologies, such as Xenium and CosMx, where only a predefined subset of genes is profiled. In this case, missing genes are excluded from score computation to avoid penalizing signatures for genes that could not be observed.

### 2.6 Parallelization

Because gene-ranking matrices are dense, their calculation may require substantial RAM. For efficient computation, UCell processes expression matrices in mini-batches, or “chunks,” typically consisting of a few hundred cells. This approach significantly reduces the method’s memory footprint, but also enables parallel execution of mini-batches over multiple threads. In the R implementation, UCell relies on BiocParallel (Morgan *et al.* 2014) for parallelization, whereas the Python implementation relies on joblib. Parallel with the default “loky” backend (Joblib Development Team 2020). With parallelization, pyUCell can process 10^5^ cells in <10 s, and half a million cells in about one minute on a standard desktop computer.

### 2.7 Signature kNN-smoothing

To mitigate the effect of single-cell data sparsity, it can be useful to smooth signature scores by a weighted average of the k nearest neighbors (kNN) of a given cell–typically calculated in PCA space. The kNN-smoothed signature score UCell_kNN_ is calculated as:


$${\text{UCell}}_{\text{kNN}}=\frac{\sum_{i=1}^{k}w_{i}\;{\text{UCell}}_{i}}{\sum_{i=1}^{k}w_{i}},\;\text{where}\;w_{i}={\left(1-\lambda \right)}^{i}$$

The decay parameter λ ∈ [0,1] modulates how much weight to assign to increasingly distant neighbors. In its extreme values, λ = 0 gives equal weight to all k neighbors, whereas λ = 1 does not apply any smoothing. Nearest neighbors are calculated using BiocNeighbors (Lun 2020) in the R implementation, and using scanpy.pp.neighbors (Wolf *et al*. 2018) in the python implementation.

## 3 Results

UCell evaluates the activity of one or several gene signatures in single-cell datasets. Gene signatures (or gene sets) are provided as lists of genes, optionally annotated with + or – suffixes to indicate inclusion in a positive or negative gene set, respectively. UCell can be applied in principle to any single-cell technology and supports inputs in SingleCellExperiment, Seurat and AnnData formats (Fig. 1A). See also Supplementary Note, available as supplementary data at *Bioinformatics* online for applications to spatial transcriptomics. Rank-based signature scores (ranging from 0 to 1) are computed independently for each cell and can be visualized in low-dimensional reductions (Fig. 1B). The resulting UCell score distributions can then be compared across intrinsic data structures such as cell clusters (Fig. 1C), or across sample-level grouping factors including experimental conditions, disease states, or treatments (Fig. 1D).

We note that directly comparing score distributions at the single-cell level may introduce pseudo-replication bias, leading to artificially inflated *P*-values (Zimmerman *et al*. 2021). We therefore recommend, whenever possible, using the sample as the unit of replication, and performing statistical tests on average UCell scores per sample (Fig. 1C and D). Another potential pitfall arises when signatures are derived from a dataset (e.g. by differential expression analysis) and subsequently used to test for differences between clusters in that same dataset. This constitutes “double dipping” and should be avoided (Squair *et al.* 2021).

To mitigate sparsity in single-cell data, UCell also allows optional smoothing of signature scores by computing a weighted average of the scores of each cell’s k-nearest neighbors (kNN). This smoothing step reduces outliers and mitigates data sparsity, producing more coherent score distributions in regions of high signature activity (Fig. 1E). In contrast to gene-level imputation, which reconstructs expression values for all genes, UCell applies kNN-smoothing solely to aggregated signature scores, improving score stability and visualization while preserving the original expression matrix.

UCell enables several downstream analyses, including cell type annotation, pathway and functional scoring, trajectory inference, and cross-dataset comparisons (Andreatta *et al*. 2022, Cook and Vanderhyden 2022, Winkler *et al.* 2022, Morabito *et al.* 2023, Liu *et al.* 2024). Its robustness and computational efficiency make it particularly well-suited to large and heterogeneous single-cell datasets. UCell v2 is available for both R and Python programming languages on BioConductor (https://bioconductor.org/packages/UCell/) and pyPI (https://pypi.org/project/pyucell/).

## Supplementary Material

btag055_Supplementary_Data

## Data Availability

No new data were generated in support of this study. Reproducible tutorials are available at https://bioconductor.org/packages/UCell/ and https://pyucell.readthedocs.io

## References

[btag055-B1] Aibar S , González-Blas CB, Moerman T et al SCENIC: single-cell regulatory network inference and clustering. Nat Methods 2017;14:1083–6. 28991892 10.1038/nmeth.4463 PMC5937676

[btag055-B2] Andreatta M , Berenstein AJ, Carmona SJ. scGate: marker-based purification of cell types from heterogeneous single-cell RNA-seq datasets. Bioinformatics 2022;38:2642–4. 35258562 10.1093/bioinformatics/btac141 PMC9048671

[btag055-B3] Andreatta M , Carmona SJ. UCell: robust and scalable single-cell gene signature scoring. Comput Struct Biotechnol J 2021;19:3796–8. 34285779 10.1016/j.csbj.2021.06.043 PMC8271111

[btag055-B4] Cook DP , Vanderhyden BC. Transcriptional census of epithelial-mesenchymal plasticity in cancer. Sci Adv 2022;8:eabi7640. 34985957 10.1126/sciadv.abi7640 PMC8730603

[btag055-B5] Fan C , Chen F, Chen Y et al irGSEA: the integration of single-cell rank-based gene set enrichment analysis. Brief Bioinf 2024;25:bbae243. 10.1093/bib/bbae243 PMC11129768 38801700

[btag055-B6] Joblib Development Team. JOBLIB: Running Python Functions as Pipeline Jobs. 2020.

[btag055-B7] Kanehisa M , Furumichi M, Tanabe M et al KEGG: new perspectives on genomes, pathways, diseases and drugs. Nucleic Acids Res 2017;45:D353–61. 27899662 10.1093/nar/gkw1092 PMC5210567

[btag055-B8] Liberzon A , Birger C, Thorvaldsdóttir H et al The molecular signatures database (MSigDB) hallmark gene set collection. Cell Syst 2015;1:417–25. 26771021 10.1016/j.cels.2015.12.004 PMC4707969

[btag055-B9] Liu ZL , Meng XY, Bao RJ et al Single cell deciphering of progression trajectories of the tumor ecosystem in head and neck cancer. Nat Commun 2024;15:2595. 38519500 10.1038/s41467-024-46912-6 PMC10959966

[btag055-B10] Luecken MD , Theis FJ. Current best practices in single-cell RNA-seq analysis: a tutorial. Mol Syst Biol 2019; 15: e8746. 31217225 10.15252/msb.20188746 PMC6582955

[btag055-B11] Lun A. BiocNeighbors: nearest neighbor detection for Bioconductor packages. R package version. 2020;1.

[btag055-B12] Mann HB , Whitney DR. On a test of whether one of two random variables is stochastically larger than the other. Ann Math Statist 1947;18:50–60.

[btag055-B13] Milacic M , Beavers D, Conley P et al The reactome pathway knowledgebase 2024. Nucleic Acids Res 2024;52:D672–8. 37941124 10.1093/nar/gkad1025 PMC10767911

[btag055-B14] Morabito S , Reese F, Rahimzadeh N et al hdWGCNA identifies co-expression networks in high-dimensional transcriptomics data. Cell Rep Methods 2023;3:100498. 37426759 10.1016/j.crmeth.2023.100498 PMC10326379

[btag055-B15] Morgan M , Obenchain V, Lang M et al BiocParallel: Bioconductor facilities for parallel evaluation. R package version. 2014;1.

[btag055-B16] Noureen N , Ye Z, Chen Y et al Signature-scoring methods developed for bulk samples are not adequate for cancer single-cell RNA sequencing data. Elife 2022;11:e71994. 35212622 10.7554/eLife.71994 PMC8916770

[btag055-B17] Squair JW , Gautier M, Kathe C et al Confronting false discoveries in single-cell differential expression. Nat Commun 2021;12:5692. 34584091 10.1038/s41467-021-25960-2 PMC8479118

[btag055-B18] Stein-O’Brien GL , Arora R, Culhane AC et al Enter the matrix: factorization uncovers knowledge from omics. Trends Genet 2018;34:790–805. 30143323 10.1016/j.tig.2018.07.003 PMC6309559

[btag055-B19] Virshup I , Bredikhin D, Heumos L et al; Scverse Community. The scverse project provides a computational ecosystem for single-cell omics data analysis. Nat Biotechnol 2023;41:604–6. 37037904 10.1038/s41587-023-01733-8

[btag055-B20] Wang RH , Thakar J. Comparative analysis of single-cell pathway scoring methods and a novel approach. NAR Genomics Bioinf 2024;6:lqae124. 10.1093/nargab/lqae124 PMC11420841 39318507

[btag055-B21] Winkler EA , Kim CN, Ross JM et al A single-cell atlas of the normal and malformed human brain vasculature. Science 2022; 375: eabi7377. 35084939 10.1126/science.abi7377 PMC8995178

[btag055-B22] Wolf FA , Angerer P, Theis FJ. SCANPY: large-scale single-cell gene expression data analysis. Genome Biol 2018;19:15. 29409532 10.1186/s13059-017-1382-0 PMC5802054

[btag055-B23] Zimmerman KD , Espeland MA, Langefeld CD. A practical solution to pseudoreplication bias in single-cell studies. Nat Commun 2021;12:738. 33531494 10.1038/s41467-021-21038-1 PMC7854630

