# Supplementary material for: UCell and pyUCell: single-cell gene signature scoring for R and Python
Source: Bioinformatics. 2026 Feb 3;42(2):btag055. doi: 10.1093/bioinformatics/btag055 (PMC12925249; doi:10.1093/bioinformatics/btag055)
Supplement: btag055_Supplementary_Data [file btag055_supplementary_data.pdf]

Supplementary Note for:

UCell and pyUCell: single-cell gene signature scoring for R and Python

Massimo Andreatta and Santiago J. Carmona

Bioinformatics (2026)

This supplementary note accompanies the manuscript "UCell and pyUCell: single-cell gene signature scoring for R and Python" and provides additional guidelines for optimal usage of UCell. We cover a few essential analysis steps, important parameters, and common user questions.

Contents:

1. Data normalization
2. Missing genes: impute or skip?
3. Positive and negative gene sets
4. From UCell scores to cell type labels
5. Analyzing and comparing multiple samples
6. UCell for spatial transcriptomics

1. Data normalization

The goal of normalization in single-cell data is to make observed measurements (raw expression counts) comparable across cells by accounting for systematic, non-biological variation. A major source of variation is sequencing depth/capture efficiency, which can differ substantially between cells and lead to large differences in total counts and number of detected genes (Stegle, Teichmann and Marioni 2015). The simplest and most widely used normalization approach is size-factor normalization, in which counts for each cell are divided by a cell-specific scaling factor. This approach assumes that cell-specific technical biases (e.g. capture efficiency) affect all genes approximately equally within a given cell. Simple size-factor normalizations such as counts per million (CPM) or counts per 10,000 (CP10k), often followed by a log1p transformation, are commonly used in single-cell analysis and have been shown to perform competitively – and in many cases outperform – more complex transformations (Ahlmann-Eltze and Huber 2023).

Because UCell scores are rank-based and computed independently for each cell, they are invariant to size-factor normalizations such as CPM, CP10k, or log1p transformations. As a result, UCell scores are identical whether computed from raw counts or from data normalized using these approaches. In contrast, model-based approaches for normalization that fit counts to a probabilistic method, such as SCTransform (Hafemeister and Satija 2019) and scran deconvolution (L. Lun, Bach and Marioni 2016), aim to stabilize variance and can be beneficial for certain downstream tasks such as clustering and data integration. However, these methods alter the relative ranking of genes within individual cells and are therefore not recommended as preprocessing for UCell.

Similarly, batch-effect correction methods – which can be viewed as a form of normalization – are often applied to remove unwanted technical variation in multi-sample or multi-study single-cell datasets (Luecken *et al.* 2022). While batch-corrected expression spaces are well suited for visualization, joint clustering, and cell-type annotation, the corrected expression values are highly distorted and should not be used for gene signature scoring. Accordingly, even when analyzing integrated reference atlases, we recommend computing UCell scores on the original, uncorrected gene expression matrices for each individual sample.

2. Missing genes: impute or skip?

A key technical consideration in gene signature scoring is how to handle genes from a signature that are absent from the input expression matrix. UCell v2 provides two strategies for handling missing genes, corresponding to different assumptions about why those genes are missing.

Option 1 – Impute missing genes as zero counts.

This option assumes that genes absent from the count matrix have zero (or below-detection) expression in the cell. This situation commonly arises in processed scRNA-seq datasets deposited in public repositories, where lowly detected genes are often filtered out during preprocessing. Under this assumption, missing genes are treated as unexpressed and contribute accordingly to the signature score. *In practice, this option is recommended for whole-transcriptome scRNA-seq data when the input matrix has undergone gene filtering and the absence of a gene is likely to reflect low or undetectable expression rather than a limitation of the assay.*

Option 2 – Skip missing genes.

This option assumes that missing genes were not measured by the assay rather than being unexpressed. This is typical of targeted or panel-based single-cell technologies, such as Xenium and CosMx, where only a predefined subset of genes is profiled. In this case, missing genes are excluded from score computation to avoid penalizing signatures for genes that could not have been observed. *In practice, this option should be used for targeted or panel-based assays, or whenever the gene universe is constrained by experimental design rather than preprocessing.*

3. Positive and negative gene sets

Gene signatures in UCell can comprise both positive and negative genes. Given two gene sets, $\mathbf{s}^+$ and $\mathbf{s}^-$, UCell scores are computed independently for each set and then combined as follows:

$$\mathrm{UCell} = \mathrm{UCell}^+ - w \cdot \mathrm{UCell}^-$$

The default value for the weighting parameter (*w=1*) assigns equal weight to the positive and negative components. Because UCell^+ and UCell^- are calculated separately, they are not affected by the number of genes in either component, and they are, by construction, bound by the [0, 1] range. Therefore, for the vast majority of applications, users will not need to change the *w* parameter. Specific cases where tuning of "w" may be desired include instances where genes in the positive set (e.g. cell type markers) are more poorly expressed than those in the negative set, or vice versa. Even in these cases, however, defining more robust signatures is preferable rather than tuning the *w* parameter.

4. From UCell scores to cell type labels

Gene signature scoring in single-cell data provides a quantitative, continuous measure of the activity of predefined biological programs or pathways. While this continuous representation is well-suited to describing graded biological processes, a distinct challenge arises when gene signatures are used for cell-type annotation. In this setting, continuous scores for cell-type marker signatures must be translated into discrete cell-type labels. A common approach is to define thresholds on signature scores and assign cell types based on whether these thresholds are exceeded. Although straightforward and effective, this strategy has several limitations. For example, cells may score highly for multiple cell-type signatures, sparsity may lead to unreliable assignments at the single-cell level, and it may be difficult to define highly specific marker signatures for all cell types – particularly at fine levels of granularity.

To address these challenges, we previously introduced scGate, a framework that automates the conversion of UCell scores into cell type assignments (Andreatta, Berenstein and Carmona 2022). scGate is based on gating models (GMs), which are collections of gene signatures that define a target cell population, conceptually analogous to gating strategies used in flow cytometry. For a given GM, scGate computes UCell scores for each constituent signature and applies k-nearest neighbor (kNN) smoothing to reduce the impact of data sparsity by propagating signal across transcriptionally similar cells. Thresholds are then applied to the smoothed scores within binary decision trees to assign cell type labels. scGate supports both sequential and hierarchical gating strategies, thereby enabling the classification of cell populations at arbitrary levels of resolution. The package includes a collection of predefined GMs, while also allowing users to define custom signatures and models. scGate is implemented in R and is available as a CRAN package (https://cran.r-project.org/package=scGate). When UCell scores are to be used for discrete cell type classification, we recommend leveraging the scGate framework.

5. Analyzing and comparing multiple samples

Single-cell research is increasingly shifting from the analysis of individual datasets toward multi-sample studies and large-scale meta-analyses comprising hundreds or thousands of samples. In the context of gene signature analysis, this transition raises important questions regarding robustness to technical and batch effects, as well as the appropriate choice of biological replicates for statistical testing.

As discussed above, UCell addresses sparsity by capping gene ranks at a maximum value defined by the parameter r_{max}. This approach restricts ranking to non-zero measurements while collapsing the long tail of zero values that is characteristic of single-cell expression data. As a practical guideline, r_{max} should be set approximately to the median number of detected (non-zero) genes per cell, thereby focusing rankings on the informative portion of each cell's expression profile. The default value (r_{max} = 1500) is appropriate for 10x Genomics Chromium data and other predominant scRNA-seq technologies. However, users should inspect the distribution of detected genes in their data and adjust r_{max} accordingly. This is particularly important for datasets with constrained feature spaces, such as probe-based spatial transcriptomics platforms (e.g. Xenium, CosMx), which typically profile only a few hundred to a few thousand genes. When jointly analyzing multiple samples, we recommend using a single r_{max} value across all samples to ensure comparability of UCell scores. In practice, this value should be chosen such that it does not substantially exceed the number of detected genes in any sample, effectively anchoring r_{max} to the lowest-quality samples in the cohort. If samples differ markedly in the number of detected genes, UCell scores may not be reliably comparable across samples. In such cases, we recommend excluding low-quality samples from downstream signature-based comparisons, when possible.

A second critical consideration in multi-sample gene signature analysis is the choice of replication unit for statistical testing. Directly comparing UCell score distributions at the single-cell level can introduce pseudo-replication bias, resulting in artificially inflated significance estimates (Zimmerman, Espeland and Langefeld 2021). When sample sizes permit, we therefore recommend using the biological sample – not individual cells – as the unit of replication, and performing statistical tests on sample-level summaries such as mean UCell scores. Finally, caution is warranted when gene signatures are derived from the same dataset in which they are subsequently tested (e.g. signatures defined by differential expression and then used to compare clusters within that dataset). This practice constitutes "double dipping" and can lead to biased inference, and should therefore be avoided (Squair *et al.* 2021).

6. UCell for spatial transcriptomics

Spatial transcriptomics technologies are becoming increasingly prevalent in the single-cell transcriptomics landscape, motivating the need for specific guidelines when applying UCell to spatial data. Conceptually, spatial transcriptomics assays generate expression matrices that are similar in structure to those commonly analyzed in scRNA-seq. However, important differences in resolution, gene coverage, and data sparsity require careful consideration, both in terms of parameter selection and interpretation of the resulting scores.

First, most spatial transcriptomics technologies do not yet achieve true single-cell resolution. For example, 10x Genomics Visium profiles gene expression on a regular grid over a tissue section, where each spatial "spot" typically captures transcripts from multiple cells, ranging from one to several tens depending on tissue density and spot size. As a consequence, UCell scores computed for Visium spots represent averaged gene signature activity across the constituent cells rather than cell-intrinsic activity. Whether this approximation is appropriate depends on the biological question, the degree of cellular heterogeneity within spots, and the spatial scale of the signatures being interrogated.

Second, several UCell parameters require adjustment when working with spatial data, particularly for probe-based or panel-based spatial technologies such as Xenium or CosMx. These platforms profile a restricted set of genes, typically on the order of a few hundred to a few thousand, making it essential to assess whether the genes in a given signature are sufficiently represented in the panel. If a substantial fraction of a signature's genes is not measured, the sensitivity and interpretability of the resulting UCell scores will be reduced, and such signatures should be interpreted with caution or excluded from analysis. As discussed above, when using targeted spatial assays, the UCell "missing genes" option should be set to "skip", to avoid imputing zero expression values for genes that were not measured by the assay. In addition, the r_{max} parameter should be adapted to the limited feature space of these technologies. Specifically, r_{max} should not exceed the total number of genes in the panel, and in practice should be set more conservatively to approximately the median number of detected (non-zero) genes per cell (or spatial unit). This is particularly important when gene detection is uneven across the panel or overall capture efficiency is low. Together, these considerations ensure that UCell scores computed on spatial transcriptomics data remain interpretable and comparable, while respecting the technical constraints and biological resolution of the underlying assays.

To illustrate with an example the application of UCell to spatial transcriptomics, we re-analyzed a dataset of basal-cell carcinoma (BCC) (Yerly *et al.* 2024) collected using the 6k-plex platform CosMx SMI and available from zenodo at: https://zenodo.org/records/14330691 . For illustrative purposes, we will use UCell to quantify two simple gene signatures for keratinocytes and fibroblasts, defined as follows:

keratinocytes = KRT5+, KRT14+, KRT1+, KRT10+, KRTDAP+

fibroblasts = COL1A1+, COL1A2+, COL3A1+, DCN+, LUM+, THY1+, KRT18-, KRT19-

Although the gene panel comprises 6000 genes, fewer than 500 genes were detected per cell on average (**Figure S1A**). Therefore, we set r_{max} = 400 to limit gene ranking to focus on the informative portion of the expression profile. It is also important to verify that the genes in the signatures are present in the panel, and/or set the “missing genes” parameter to “skip” to avoid imputing zero expression for missing genes. Visualizing UCell scores for fibroblasts and keratinocytes in spatial coordinates for representative fields of view (FOV) shows how they correspond to different spatial regions, highlighting the architecture of the tissue (**Figure S1B**).

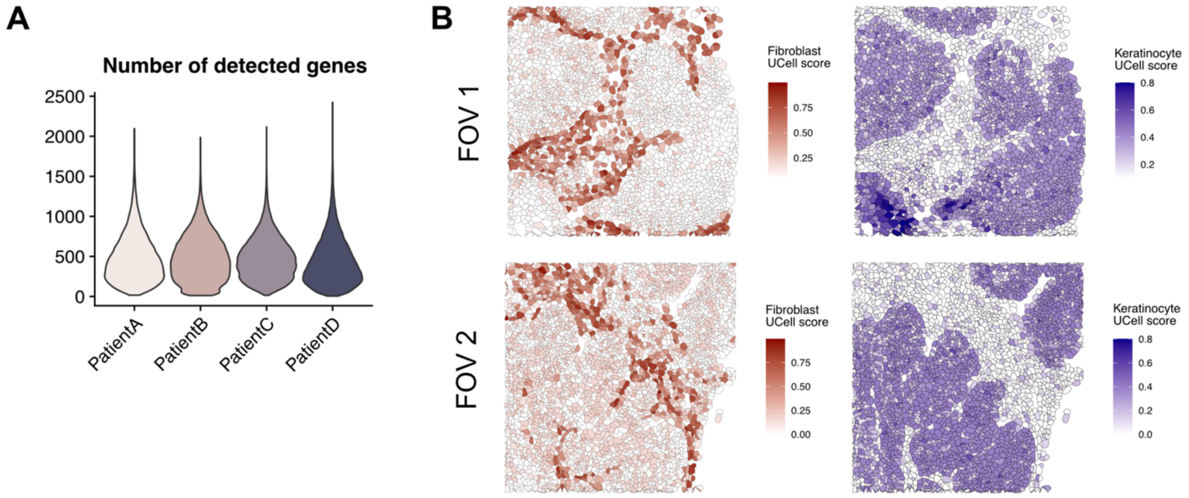

Figure S1: Example analysis of spatial transcriptomics data with UCell. A) Number of detected genes per patient in a CosMx 6-plex dataset of BCC. **B)** UCell scores for two gene signatures (fibroblasts and keratinocytes) in two representative fields of view (FOV1 and FOV2).

References

Ahlmann-Eltze C, Huber W. Comparison of transformations for single-cell RNA-seq data. *Nat Methods* 2023;**20**:665–72.

Andreatta M, Berenstein AJ, Carmona SJ. scGate: marker-based purification of cell types from heterogeneous single-cell RNA-seq datasets. *Bioinformatics* 2022;**38**:2642–4.

Hafemeister C, Satija R. Normalization and variance stabilization of single-cell RNA-seq data using regularized negative binomial regression. *Genome Biol* 2019;**20**:296.

L. Lun AT, Bach K, Marioni JC. Pooling across cells to normalize single-cell RNA sequencing data with many zero counts. *Genome Biol* 2016;**17**:75.

Luecken MD, Büttner M, Chaichoompu K *et al.* Benchmarking atlas-level data integration in single-cell genomics. *Nat Methods* 2022;**19**:41–50.

Squair JW, Gautier M, Kathe C *et al.* Confronting false discoveries in single-cell differential expression. *Nature communications* 2021;**12**:5692.

Stegle O, Teichmann SA, Marioni JC. Computational and analytical challenges in single-cell transcriptomics. *Nat Rev Genet* 2015;**16**:133–45.

Yerly L, Andreatta M, Garnica J *et al.* Wounding triggers invasive progression in human basal cell carcinoma. 2024, DOI: 10.1101/2024.05.31.596823.

Zimmerman KD, Espeland MA, Langefeld CD. A practical solution to pseudoreplication bias in single-cell studies. *Nature communications* 2021;**12**:738.
